# Effects of *Lactobacillus plantarum* and *Lactobacillus paracasei* on the Peripheral Immune Response in Children with Celiac Disease Autoimmunity: A Randomized, Double-Blind, Placebo-Controlled Clinical Trial

**DOI:** 10.3390/nu11081925

**Published:** 2019-08-16

**Authors:** Åsa Håkansson, Carin Andrén Aronsson, Charlotte Brundin, Elin Oscarsson, Göran Molin, Daniel Agardh

**Affiliations:** 1 Department of Food Technology Engineering and Nutrition, Lund University, Box 124, 22100 Lund, Sweden; 2 The Diabetes and Celiac Disease Unit, Department of Clinical Sciences, Lund University, 21428 Malmö, Sweden

**Keywords:** celiac disease, probiotic, autoimmunity, immune function

## Abstract

Two *Lactobacillus* strains have proven anti-inflammatory properties by reducing pro-inflammatory responses to antigens. This randomized double-blind placebo-controlled trial tested the hypothesis that *L. plantarum* HEAL9 and *L. paracasei* 8700:2 suppress ongoing celiac disease autoimmunity in genetically at risk children on a gluten-containing diet in a longitudinally screening study for celiac disease. Seventy-eight children with celiac disease autoimmunity participated of whom 40 received 10^10^ CFU/day of *L. plantarum* HEAL9 and *L. paracasei* 8700:2 (probiotic group) and 38 children maltodextrin (placebo group) for six months. Blood samples were drawn at zero, three and six months and phenotyping of peripheral blood lymphocytes and IgA and IgG autoantibodies against tissue transglutaminase (tTG) were measured. In the placebo group, naïve CD45RA+ Th cells decreased (*p* = 0.002) whereas effector and memory CD45RO+ Th cells increased (*p* = 0.003). In contrast, populations of cells expressing CD4+CD25^high^CD45RO+CCR4+ increased in the placebo group (*p* = 0.001). Changes between the groups were observed for NK cells (*p* = 0.038) and NKT cells (*p* = 0.008). Median levels of IgA-tTG decreased more significantly over time in the probiotic (*p* = 0.013) than in the placebo (*p* = 0.043) group whereas the opposite was true for IgG-tTG (*p* = 0.062 respective *p* = 0.008). In conclusion, daily oral administration of *L. plantarum* HEAL9 and *L. paracasei* 8700:2 modulate the peripheral immune response in children with celiac disease autoimmunity.

## 1. Introduction

Changes in the intestinal microbiota and dysregulation of the mucosal immune response may contribute to the pathogenesis of different inflammatory and autoimmune disorders. Probiotics, defined as live microorganisms which when administrated in adequate amounts confer a health benefit [1], are known to have immunomodulatory influences on leucocyte populations and lymphocyte phenotypes [2], the ability to promote the endogenous defense barrier in the human gut [3], and the ability to decrease mucosal permeability [4].

Asthma and food allergies were the first diseases where probiotics had proven anti-inflammatory effects [5]. Subsequent studies have shown that probiotics can reduce autoimmune responses in patients with rheumatoid arthritis by improving inflammatory status and disease activity [6]. Furthermore, the risk of islet autoimmunity was reduced by probiotics in children at the highest genetic risk of type 1 diabetes [7], and probiotics have induced and maintained remission in children with ulcerative colitis [8]. Although further studies are required to evaluate reproducibility and health promoting effects of probiotics in autoimmune diseases, several larger meta-analyses have actually shown improvements of clinical conditions [9,10,11]. However, the effects of probiotics are notwithstanding highly dependent on what type of microorganism that is administrated. For instance, the present combination of two *Lactobacillus* strains are chosen due to their different physiological effects, i.e., *L. plantarum* HEAL9 is targeting the permeability of the mucosa and *L. paracasei* 8700:2 is targeting the immune system [12,13,14,15]. 

Celiac disease is a chronic small bowel enteropathy triggered by ingestion of dietary gluten or related prolamins in genetically susceptible individuals. Celiac disease is a T cell mediated disease involving CD4+/CD8+ T cells against gliadin peptides as well as production of circulating regulatory T cells and NK cells [16,17,18,19,20]. The infiltration of HLA-DQ2 or HLA-DQ8 restricted gliadin-specific CD4+ and CD8+ lymphocytes in the intestinal mucosa [17,18] lead to destruction of the intestinal villi and increased intestinal permeability [21]. Another striking feature of celiac disease is the autoimmune response against tissue transglutaminase (tTG); a calcium-dependent intracellular enzyme involved in posttranslational deamidation of proteins [22]. Elevated levels of tTG autoantibodies indicate active celiac disease autoimmunity (CDA) whereas treatment with a gluten-free diet reverse the levels to normal over time [23]. 

Few studies have evaluated alternative treatments to a gluten-free diet and two prevention studies introducing small amounts of gluten have hitherto been unsuccessful in preventing the disease [24,25]. Probiotics are appealing due to their proven anti-inflammatory effects in other chronic diseases and low risk of side effects. Although a Finnish clinical double-blind randomized placebo controlled trial primarily on allergy prevention could not find any protective effects of probiotics in celiac disease [26], randomized trials in genetically at risk individuals before the disease onset are still warranted. 

The purpose of the present randomized double-blind placebo-controlled study was to evaluate the immunomodulatory properties of two probiotic strains of lactobacilli in children with active naïve CDA prior to diagnosis and treatment with a gluten-free diet. We tested the hypothesis that the supplement had a dampening effect on CDA as compared to the placebo by analyzing a broad panel of lymphocyte subpopulations as well as tTG autoantibody levels to receive a wide understanding of the immunological reactions caused by daily oral administration of *L. plantarum* HEAL9 and *L. paracasei* 8700:2 for six months, compared to placebo. 

## 2. Materials and Methods

### 2.1. Study Design and Participants

Children at genetic risk for celiac disease and on a gluten-containing diet who were screened positive for tTG autoantibodies in a prospective birth cohort study [27] were invited to the randomized double-blind placebo controlled clinical trial. The inclusion criteria were children on a gluten-containing diet with no previous diagnosis of celiac disease but with a tTG autoantibody level above the cutoff of normal (normal < 1.31 U/mL) in two consecutive samples taken at least three months apart, here used as the definition of CDA. The levels of tTG autoantibodies were measured in radioligand binding assays as previously described [28,29]. Using this cut-off level of normal, the assay previously achieved 100% sensitivity and specificity in the first International Transglutaminase Autoantibody Workshop [30].

A total of 118 children met the inclusion criteria and were invited to participate in the Celiac Disease Prevention with Probiotics (CiPP) study; a double-blind placebo controlled randomized clinical trial with the aim to test the effects of probiotics on the peripheral immune response in CDA, performed at the Department of Clinical Sciences, Unit of Diabetes and Celiac disease, Lund University, Malmö, Sweden. Among the invited children, 89 accepted participation and were enrolled in the study between 12 March 2012 and 25 August 2015. Of those children, 11 of 89 (12%) left the study after the initial visit (dropout). The main reasons for study dropout were low compliance (the child did not want to eat the food when the powder was included; parent forgot to give the child the powder) and symptoms such as diarrhea or vomiting. One child was also excluded due to insufficient blood sample volumes. A total of 78 children (88%) completed the study (visit three between 1 October 2012 and 26 January 2016) and were included in the final data set; 40 (52%) in the probiotic-treated group and 38 (48%) in the placebo group of whom 55% of the probiotic group and 37% of the placebo group were males (Table 1). All participants carried any of the following HLA haplotypes: DR3-DQ2/DR3-DQ2 (*n* = 35), DR3-DQ2/DR4-DQ8 (*n* = 28), DR4-DQ8/DR4-DQ8 (*n* = 18), DR4-DQ8/DR8/DQ4 (*n* = 7), and/or DR4/DR1 (*n* = 1), (Table 2).

Written informed consent was in all cases provided by the primary guardian of the child. Before agreeing to the participation, caretakers were provided written information about the study and the contact information of the study coordinator. In accordance to the Declaration of Helsinki and Swedish law, the assent of all participants was also required (to the degree their age made it possible). While all participants were recruited from other studies currently active at Skåne University Hospital (SUS), participation in these studies was not inter-connected; participants could freely, at any time, leave the study while maintaining participation in the study they were recruited from. With informed consent provided, and no established treatments currently available to reduce the risk of CDA progressing to celiac disease, the use of a placebo arm was considered non-controversial. All children were followed longitudinally by a pediatric gastroenterologist. The decision whether to perform an intestinal biopsy was based on the decision of the pediatric gastroenterologist and occurred outside the study protocol. Throughout the experimental period, all children continued eating a regular diet containing gluten. The Ethics Committee of the Medical Faculty, Lund University, approved the study on 8 September 2011 (Dnr 2011/335). The study was registered at ClinicalTrials.gov (NCT03176095). The study protocol, the Consort flow diagram, the Consort 2010 checklist and raw research data are given in Figure S1, File S1, File S2, File S3 and the File S4, respectively. The authors confirm that all ongoing and related trials for this drug/intervention are registered.

### 2.2. Follow-Up Procedures

Children matching the inclusion criteria were invited for an initial meeting and scheduled for follow-up visits approximately three and six months later (visit one (V1) at the baseline, visit two (V2) at three months post-baseline, visit three (V3) at six months post-baseline). At every visit, 10 mL of venous blood was collected for phenotyping of peripheral blood lymphocytes and for analysis of tTG autoantibodies in the serum. Demographics and anthropometry measures were registered and a possible diagnosis of celiac disease was recorded as well as adverse events. All raw research data is given in the S3 File. Participants were randomized at a 1:1 ratio to treatment or control group and the allocation was blinded to participants, clinicians and lab personnel. 

### 2.3. Study Product

The study products, provided by Probi AB and prepared in sachets, were handed out at the V1 and V2 visits. The participants were instructed to halt consumption of any other food products containing probiotics, to store the sachets refrigerated (2 °C–8 °C) and to ingest the powder after dissolution in 100 mL of cold liquid or after mixing with fruit/food, in association with a meal once daily, for a total period of six months. The parents were also instructed not to add the powder to hot drinks or hot food. The control product contained 1.0 g maltodextrin (Glucidex IT-19, Roquette, Lesterrand, France) together with yeast peptone (HYP-A, BioSpringer, Maisons-Alfort, France) and the probiotic product contained 1.0 g of maltodextrin and lyophilized bacteria (*L. plantarum* HEAL9 (DSM 15312) and *L. paracasei* 8700:2 (DSM 13434), at a total dose of 10^10^ CFU/sachet). The two test products were identical in appearance and taste. Storage stability of the probiotic product was analyzed throughout the study. 

### 2.4. Staining for Flow Cytometry and Lymphocyte Gating

Staining for flow cytometry was performed on peripheral blood mononuclear cells (PBMC) within 24 h of sampling. PBMC were separated from whole blood by a density gradient centrifugation (1800 × *g*) using a hydrophilic polysaccharide (BD Vac^®^ CPT™ Cell Preparation Tube NC FICOLL™, Becton Dickinson, NJ, USA). The aspirated interphase mononuclear layer where washed three times with RPMI-1640 (Medium w L-Glutamine, GIBCO, Thermo Fisher Scientific, Gothenburg, Sweden). The cells where counted in an Abbott (CELL_DYN Ruby) and diluted to the final concentration 1–4 × 10^6^/mL in RPMI-1640 whereupon they were stained directly with various fluorochrome-conjugated antibodies; fluorescein isothiocyanate (FITC), phycoerythrin (PE), peridinin chlorophyll protein (PerCP) and allophycocyanin (APC), directed against the following markers: CCR9 (APC) (R&D Systems, Inc, Abingdon, England), CD45RO (APC), CD62L (APC), CD25 (FITC), CD3 (FITC), CD38 (FITC), CCR4 (PE), CD8 (PE), CD45RA (PE), Integrin beta7 (PE), CD19 (PerCP), CD4 (PerCP), CD8 (PerCP), CD16+56 (PE), (all from BD Biosciences, Mississauga, CA, USA). The stained lymphocytes were acquired in a four-colour FACSCalibur^®^ (Becton Dickinson, Franklin Lake, NJ, USA) and analyzed using the CellQuestPro^®^ software (Becton Dickinson, Franklin Lake, NJ, USA). Isotype-matched control antibodies (IgG2/IgG1/CD4 isotype (FITC, PE and PerCP-Cy 5.5) and IgG1 isotype (APC), BD biosciences, CA, USA) were used to set the dot plot quadrant and calculate the percent of lymphocyte populations through subtraction of contaminating non-lymphocytes. Lymphocytes were gated based on forward- (FSC) and side scatter (SSC) and presented as percentage gated. Subgroups of T cells, CD4+ (Th) or CD8+ (Tc) cells were gated from the lymphocyte gate. From these gates, naïve cells were gated as CD3+CD4/CD8+CD45RA+CD45RO− and memory cells as CD3+CD4/CD8+CD45RA-CD45RO+. Activated and differentiated effector and memory cells were gated as CD3+CD4/8+CD62L+, CD4+CD25+CD45RA+CD45RO+, CD4+CD25+CCR4+CD45RO, CD4+CD25+CCR4+CD62L+, CD8+CD45RA+CCR9+β7+, CD3+CD4+/CD8+β7+CCR9+, and CD4+CD38+β7+CD62−. To analyze B cells, CD3+ cells were gated from the lymphocyte gate. From this gate B cells were gated as CD3+CD19+ cells. From the lymphocyte gate, CD4+ cells were gated followed by gating for CD25+ cells. This population was then examined for the expression of FoxP3+ cells. From the CD4+CD25+ gate, the percent with the highest CD25 expression, CD4+CD25^high^ were determined. The CD4+CD25^high^ lymphocyte population was then examined further for the expression of FoxP3. NK cells were gated from the lymphocyte gate. From this gate NK and NKT cells were gated as CD3-CD16+/CD56+ cells and CD3+CD16+/CD56+ respectively. 

### 2.5. Detection of Tissue Transglutaminase (tTG) Autoantibody Levels 

A radioligand binding assay (RBA) were used to assess the changes in IgA-tTG and IgG-tTG levels (U/mL) separately, as previously described [31]. In short, human tissue transglutaminase (tTG) was synthesized by in vitro transcription and translation of cDNA using the TNT SP6 Coupled Reticulocyte Lysate System (Promega, Madison, WI, USA) in the presence of 35S-methionine (Perkin Elmer, Boston, MA, USA). Both IgG-tTG and IgA-tTG were analyzed. For the IgG-tTG analysis, 35S-tTG was diluted and added to human serum and incubated overnight at 4 °C. Protein A sepharose (PAS) (Invitrogen, Thermo Fisher Scientific, Carlsbad, CA, USA) was used to separate free and antibody-bound 35S-tTG by binding IgG in the serum. The IgA-tTG analysis was performed similarly, except goat anti-human IgA-agarose (Sigma-Aldrich, St Louis, MO, USA) was used instead of the PAS. The levels of tTG autoantibodies were calculated from standard curves containing approximately 2 U/mL, 4 U/mL, 8 U/mL, 16 U/mL, 31 U/mL, 63 U/mL, 125 U/mL, 250 U/mL, 500 U/mL and 1000 U/mL of respective IgA-tTG and IgG-tTG. 

### 2.6. Statistical Analysis

The primary endpoints were changes in autoantibodies and regulatory T cells in the probiotic group compared to the placebo group assessed as changes in the peripheral immune response of B cells, NK and NKT cells, subpopulations of regulatory T cells and changes in serum levels of tTG autoantibodies after six months. All values are presented as medians to avoid the effect of extreme values. Comparison between groups of binary variables was done by means of the Fisher’s exact test. Comparison between groups on continuous and ordered categorical data was done by the Wilcoxon Rank Sum test. Due to large inter-individual variations but stable intra-individual values comparison within groups over time is of main importance. Comparison of the change over time within each group was done by means of the Wilcoxon signed rank test for continuous variables. The statistical analyses plan was that an “intention-to-treat analysis” and a “per-protocol analysis” (PP) should be performed. However, it appeared that only a per-protocol analysis was possible to perform as the patients excluded from the PP analysis did not have any data post randomization and the intention was to not impute any missing data. This reflects 11 patients out of 89, and there was no reason to suspect that the reason for study withdrawal was related to the randomized treatment. All presented *p*-values were nominal (i.e., not adjusted for multiplicity) and two-sided. Missing data were not imputed, i.e., the analyses are on observed cases. Statistics were calculated by mean of the StatXact version 10.1. 

## 3. Results

### 3.1. Demographics and Anthropometry Data

There were no difference in the anthropometry measures between the two groups (data not shown). Three children in the probiotic group and four children in the placebo group reported adverse events during the study (pain, flatulence or diarrhea) (*p* = 0.645) and one child in each group had gastrointestinal symptoms (*p* = 0.971). Six children in the probiotic group and four children in the placebo group used antibiotics during the study (*p* = 0.561). As of 1 February 2019, biopsy-proven celiac disease was diagnosed in six children (15%) in the probiotic group and five children (13%) in the placebo group after the study ended (*p* = 0.818). 

### 3.2. Changes in Peripheral Lymphocyte Subsets 

In the placebo group, the proportion of gated Th cells (CD3+CD4+) decreased a median 2.79 (IQR −5.79–0.21) %-unit (*p* = 0.019) and T_C_ cells (CD3+CD8+) increased a median 1.12 (IQR 0.12–3.49) %-unit (*p* = 0.017) only after three months. After six months, CD3+CD4+ cells had decreased a median 4.07 (IQR −9.65–1.14) %-unit (*p* = 0.039), CD3+CD4+CD62L^low^+ cells decreased, albeit not significantly, a median 0.83 (IQR −1.91–0.49) %-units (*p* = 0.051) and CD3+CD8+CD62L^low^+ cells decreased a median 0.85 (range −1.52–0.30) %-units (*p* = 0.014) (Table 3). After six months, the population of naïve Th cells (CD4+CD45RA+CD45RO-) had similarly decreased a median 4.03 (IQR −8.75–0.88) %-units (*p* = 0.002) in the placebo group. In contrast, the population of memory Th cells (CD4+CD45RA-CD45RO+) increased a median 3.82 (IQR −0.37–7.08) %-units (*p* = 0.003). Memory Th cells expressing CCR4 (CD4+CD45RO+CCR4+) increased a median 5.86 (IQR 1.00–16.46) %-units in the placebo group (*p* = 0.003). Moreover, CD4+CD25+ cells increased a median 1.37 (IQR 0.01–3.06) %-units (*p* = 0.012), naïve CD4+CD25+CD45RA+ cells decreased a median 3.76 (IQR −13.51–2.30) %-units (*p* = 0.018), whereas memory CD4+CD25+CD45RO+ cells likewise CD4+CD25+CD45RO+CCR4+ increased a median 12.56 (IQR 4.70–18.53) %-units (*p* = 0.001) and a median 6.65 (IQR 2.83–12.86) %-units (*p* = 0.001), in the same group, respectively. Naive cells (CD8+CD45RA+CD45RO-) decreased in the placebo group a median 5.57 (IQR 3.65–9.36) %-units (*p* = 0.030). In the probiotic group over time, the population of cells remained constant and the proportion of total B cells did not change in either group. 

After six months, CD4+CD25^high^+ cells increased in the placebo group a median 1.06 (IQR −0.27–1.70) %-units (*p* = 0.008) and CD4+CD25^high^+ cells expressing CD45RO or co-expressing CD45RO and CCR4 (CD4+CD25^high^+CD45RO+) increased a median 7.78 (IQR −0.50–18.90) %-units (*p* = 0.012) and a median 7.88 (IQR 0.19–15.87) %-units (*p* = 0.001) respectively. When the population of regulatory T cells were analyzed as the percentage of total number of live cells (of all CD4+ cells), CD4+CD25+FoxP3+ cells increased a median 0.31 (IQR −0.19–0.75) %-units in the placebo group, albeit not significantly (*p* = 0.052). In contrast, the proportion of all subpopulations of CD4+CD25^high^+ cells remained unchanged in the probiotic group. Furthermore, the proportion of NKT cells (CD3+CD56+) decreased a median 0.16 (IQR −0.34–0.11) %-units only in the probiotic group (*p* = 0.03), whereas the CD3+CD56+ cells remained constant in the placebo group.

When comparing probiotic versus placebo, differences in changes in cell populations expressing CD3-CD56+ (*p* = 0.038) as well as cells expressing CD3+CD56+ (*p* = 0.008) were observed (Table 3). Additionally, changes in cell populations of naïve Th cells (CD3+CD4+CD45RA+CD45RO-) (*p* = 0.022), memory Th cells (CD3+CD4+CD45RA-CD45RO+) (*p* = 0.020) and memory Th cells also expressing CCR4 (CD4+CCR4+CD45RO+) (*p* = 0.011) after six months were observed. Furthermore, differences in changes in cell populations expressing either CD4+CD25+CD45RO+ (*p* = 0.003), CD4+CD25^high^+CCR4+CD45RO+ (*p* = 0.045), CD3+CD4+CCR9+β7+ (*p* = 0.038) or CD3+CD4+CD25+Foxp3+ (*p* = 0.028) could be seen between the two groups after six months of probiotic or placebo consumption (Table 3).

### 3.3. Changes in tTG Autoantibody Levels

There was no difference in the number of tTG positive children between the groups. At the baseline visit after enrollment, 33 remained positive for IgA-tTG and 26 children positive for IgG-tTG in the probiotic group compared with 31 and 23 children in the placebo group, respectively. At the six-month visit, 23 remained positive for IgA-tTG and 24 children remained positive for IgG-tTG in the probiotic group compared with 25 and 18 children in the placebo group, respectively. There was no difference in the IgA-tTG or IgG-tTG levels between the groups after three months (*p* = 0.362 and 0.925, respectively) or after six months (*p* = 0.838 and 0.766, respectively). Neither were there any differences in the IgA-tTG levels within groups after three months (*p* = 0.134 in the probiotic group and *p* = 0.521 in the placebo group). As compared to baseline levels, IgG-tTG decreased a median 0.26 (IQR −0.56–0.18) U/mL in the probiotic group (*p* = 0.046) and a median 0.23 (IQR −0.82–0.18) U/mL in the placebo group (*p* = 0.034), respectively. After six months, levels of IgA-tTG had decreased a median 0.85 (IQR −3.30–0.24) U/mL (*p* = 0.013) and IgG-tTG decreased a median 0.29 (IQR −1.31–0.40) U/mL (*p* = 0.062) in the probiotic group. In the placebo group and compared to the baseline, levels of IgA-tTG decreased a median 0.79 (IQR −3.43–0.08) U/mL (*p* = 0.043) and IgG-tTG decreased a median 0.36 (IQR −1.12–−0.05) U/mL (*p* = 0.008). 

## 4. Discussion 

The present exploratory, randomized, double-blind, placebo-controlled trial evaluated a probiotic food supplement containing selected *Lactobacillus* strains of two functionally different species, *L. plantarum* (strain HEAL9) and *L. paracasei* (strain 8700:2). Both *Lactobacillus* strains have been previously isolated from the gastro-intestinal mucosa of healthy humans, and have the ability to function in the intestinal environment [32,33]. *L. plantarum* HEAL9 has a pronounced ability to attach to the human mucosa through a mannose-binding, adherence mechanism [32], and *L. paracasei* 8700:2 also express this function [34]. In addition, *L. plantarum* HEAL9 is genetically similar to the well-studied strain *L. plantarum* 299v, which reduces intestinal permeability probably as a result of adherence and decreased inflammation [12,13,14]. Furthermore, *L. paracasei* 8700:2 can induce cell-mediated immune functions in healthy volunteers, as it expands the NKT cell population [15]. By affecting cellular immune responses and possibly mucosal condition, oral intake of *L. plantarum* HEAL9 and *L. paracasei* 8700:2 have earlier been shown to alleviate symptoms and the duration of common colds and to reduce the risk of infections [34,35]. 

The main finding of the present study was the consistent changes in the peripheral immune response involved in the regulation of T cells only observed in the placebo group and not in the probiotic group despite that both groups had ongoing CDA before intervention. This finding of importance indicates that *L. plantarum* (strain HEAL9) and *L. paracasei* (strain 8700:2) have modulatory effects on the peripheral immune response in CDA. Interestingly, the difference of most lymphocyte subsets found in the placebo group was similar to what is found in patients with active celiac disease, indicating a progression of disease development not observed in the probiotic group. The increase of CD4+CD25+Foxp3+ T cells in the placebo group, which remained unchanged in the probiotic group and clearly showed a difference between the two groups, could be explained by the down regulatory effects of the two *Lactobacillus* strains on activated CD4+ cells. The observed reduction in CD3+CD4+ cells in the placebo group may be considered as secondary to the compartmentalization of gluten-sensitive lymphocytes within the intestinal mucosa. Furthermore, naïve Th cells (CD45RA+) were reduced meanwhile the percentage of effector and memory Th cells (CD45RO+) was higher in the placebo group, which has previously been observed in untreated celiac disease patients and explained by higher percentages of circulating CD45+ αβTcR+ and γδTcR+ lymphocytes activated by gluten [36]. This explanation is further strengthened by the finding of an increased percentage of CD45RO+ cells also expressing CCR4 in the placebo group, suggesting a re-circulation of primed regulatory T cells. CCR4 is an important chemokine receptor for recruitment of T cells to the sight of inflammation and it is highly expressed on differentiated regulatory T cells [37]. The increase of CD4+CD25^high^CD45RO+CCR4+ cells as well as CD4+CD25+Foxp3+ cells in the placebo group indicate an attempt to extinguish an ongoing intestinal inflammation and the immune response to dietary gluten antigens as previously described [19].

Another finding of importance was the peripheral changes in NKT cells over time in the probiotic group, which was not observed in the placebo-children. The population of NK and NKT cells has been found to decrease in both tissue and periphery in active celiac disease, however contradictory results have been found in comparison between adults and children [38]. Moreover, NK cells can function by promoting or even protecting against the onset of autoimmune conditions and in patients at risk of developing celiac disease the absolute count of NK cells was significantly higher in intermediate risk patients than in high-risk and low-risk patients [39]. 

In contrast to the peripheral immune response, the effects on the tTG autoantibody levels in both groups and for respective Ig isotype after intervention were less clear. There was overall no difference in the median levels of the tTG autoantibody between the two groups over time, albeit the decrease in levels were stronger for IgA-tTG than that of IgG-tTG. It is well known that the IgA-tTG levels decrease more rapidly than that of IgG-tTG in celiac disease patients treated with a gluten-free diet [34], and it is possible that the same phenomenon could be observed after probiotic consumption. Another reason for the divergent results could be due to a reduced power of the study to find differences between participants detected in the outer ranges of low and high tTG autoantibody levels. Using CDA and not celiac disease as an endpoint could be considered as another limitation of the study. Not all individuals with CDA necessarily develop celiac disease although the vast majority of patients have CDA prior to or at diagnosis. Furthermore, the intervention of this study was for a rather short and limited time. Therefore, this study cannot conclude that probiotics may prevent celiac disease, or have proven effects on the intestinal mucosa, albeit a peripheral response was significant already after six months of intervention. Indeed, the long-term effects of probiotics if given continuously over an extended period of time would be valuable. 

## 5. Conclusions

This randomized, double-blind, placebo-controlled study found that a daily oral administration of *L. plantarum* HEAL9 and *L. paracasei* 8700:2 may modulate the peripheral immune response in children with CDA. These findings need to be evaluated in larger longer follow-up studies before any potential preventive effects of *Lactobacillus* on the development of celiac disease can be attributed. 

## 6. Patents

D. Agardh (M.D., Ph.D.) is stated as inventor in a patent application based on the results of the clinical trial but have signed over all legal rights to the patent to Probi AB.

## Figures and Tables

**Table 1:** Demographics and study-specific measures at the baseline visit. Measures are presented as the median and interquartile range (IQR), Q1–Q3.

Variable	Probiotic Group	Placebo Group	*p*-Value ^1^
Age (years, median (IQR))	5 (3–7)	4 (3–6)	0.284
Weight (kg, median (IQR))	21 (18–25)	20 (17–22)	0.182
Length (cm, median (IQR))	116 (102–126)	109 (102–117)	0.243
IgA-tTG (U/mL, median (IQR))^2^	4.71 (1.58–12.21)	4.38 (1.90–13.19)	0.891
IgG-tTG (U/mL, median (IQR)) ^2^	1.57 (1.11–4.74)	1.60 (1.24–4.59)	0.848
First degree relative diagnosed with celiac disease (*n* (%))	6 (15.0)	3 (7.9)	0.334
Use of foods/supplements fortified with probiotics before study start (*n* (%))	19 (48)	18 (47)	0.995

^1^ Wilcoxon signed rank test. Two-sided. ^2^ tTG -tissue transglutaminase.

**Table 2:** Human leucocyte antigen (HLA) distribution among the 78 children that completed the study.

HLA-Type	Probiotic Group n (%)	Placebo Group *n* (%)	*p*-Value ^1^
DR3-DQ2/DR3-DQ2	15 (37.5)	13 (34.2)	0.8162
DR3-DQ2/DR4-DQ8	10 (25.0)	16 (42.1)	0.1500
DR4-DQ8/DR4-DQ8	10 (25.0)	7 (18.4)	0.5869
DR4-DQ8/DR8/DQ4	4 (10.0)	2 (5.3)	0.6755
DR4/DR12	1 (2.5)	0 (0.0)	1.000

^1^ Fisher’s exact test. Two-sided *p*-value.

**Table 3:** Absolute changes in lymphocyte subsets within the groups and between groups after six months presented as median (IQR) percentage gated cells and median (IQR) percentage of total cell.

Study Outcome	Probiotic Group				Placebo Group				Probiotic Group vs. Placebo Group
	0 Months	6 Months	0 vs. 6 Months	*p*-Value	0 Months	6 Months	0 vs. 6 Months	*p*-Value	*p*-Value
Lymphocyte Subpopulation (Median (IQR) % Unit)									
Gated									
0	8.79 (5.22–11.84)	7.07 (5.56–9.47)	−1.61 (−4.37–2.53)	0.264	7.18 (4.63–9.98)	7.65 (5.75–13.09)	+2.18 (−1.99–6.14)	0.061	0.038
CD3+CD56+	0.44 (0.37–0.79)	0.44 (0.23–0.69)	−0.16 (−0.34–0.11)	0.03	0.46 (0.32–0.69)	0.58 (0.45–0.88)	+0.18 (−0.11–0.38)	0.108	0.008
CD3+CD4+CD45RA+ CD45RO-	68.60 (60.83–71.05)	65.01 (62.70–71.12)	−0.59 (−3.76–4.08)	0.746	71.52 (64.93–75.27)	68.87 (58.61–71.14)	−4.03 (−8.75−0.88)	0.002	0.022
CD3+CD4+CD45RA-CD45RO+	18.46 (13.64–23.55)	18.40 (14.12–22.06)	+0.05 (−4.32–3.32)	0.851	14.89 (11.85–19.43)	17.85 (13.06–21.56)	+3.82 (−0.37–7.08)	0.003	0.02
CD4+CCR4+CD45RO+	65.03 (59.52–72.07)	66.43 (61.31–74.79)	+0.50 (−5.79–6.98)	1	65.73 (56.81–72.67)	69.99 (65.23–77.56)	+5.86 (1.00–16.46)	0.003	0.011
CD4+CD25+CD45RA+	28.76 (20.95–39.98)	30.19 (23.73–39.65)	−0.80 (−8.39–3.15)	0.522	39.71 (29.77–44.41)	35.89 (19.59–41.02)	−3.76 (−13.51–2.30)	0.018	0.193
CD4+CD25+CD45RO+	49.80 (38.49–62.09)	49.82 (42.48–59.20)	+4.67 (−6.18–8.87)	0.416	44.76 (33.99–51.10)	48.32 (41.49–65.68)	+12.56 (4.70–18.53)	0.0006	0.003
CD4+CD25+CCR4+ CD45RO+	70.63 (64.65–77.49)	72.53 (65.35–81.45)	+2.40 (−3.74–11.31)	0.237	68.36 (60.54–75.96)	75.77 (70.7–81.39)	+6.65 (2.83–12.86)	0.0007	0.083
CD4+CD25^high^+CCR4+CD45RO+	75.68 (67.80–85.28)	78.81 (68.20–83.74)	+3.97 (−4.42–7.85)	0.326	73.76 (63.43–82.79)	82.87 (77.06–89.13)	+7.88 (0.19–15.87)	0.001	0.045
CD3+CD4+CCR9+β7+	1.34 (1.01–1.94)	1.46 (1.16–2.05)	+0.21 (−0.44–0.40)	0.623	1.65 (1.21–2.35)	1.42 (1.06–2.13)	−0.33 (−1.06–0.09)	0.024	0.038
CD4+CD25+	8.82 (7.23–12.44)	9.11 (7.58–11.18)	−0.10 (−3.31–2.51)	0.839	9.15 (7.14–12.51)	9.88 (7.63–12.37)	+1.37 (0.01–3.06)	0.012	0.1
CD4+CD25^high^	3.93 (3.10–5.34)	4.30 (3.12–4.96)	+0.08 (−1.41–1.55)	0.867	4.04 (2.99–5.21)	4.63 (3.43–5.54)	+1.06 (−0.27–1.7)	0.008	0.103
0	8.79 (5.22–11.84)	7.07 (5.56–9.47)	−1.61 (−4.37–2.53)	0.264	7.18 (4.63–9.98)	7.65 (5.75–13.09)	+2.18 (−1.99–6.14)	0.061	0.038
CD3+CD56+	0.44 (0.37–0.79)	0.44 (0.23–0.69)	−0.16 (−0.34–0.11)	0.03	0.46 (0.32–0.69)	0.58 (0.45–0.88)	+0.18 (−0.11–0.38)	0.108	0.008
CD3+CD4+CD45RA+ CD45RO-	68.60 (60.83–71.05)	65.01 (62.70–71.12)	−0.59 (−3.76–4.08)	0.746	71.52 (64.93–75.27)	68.87 (58.61–71.14)	−4.03 (−8.75-−0.88)	0.002	0.022
CD3+CD4+CD45RA-CD45RO+	18.46 (13.64–23.55)	18.40 (14.12–22.06)	+0.05 (−4.32–3.32)	0.851	14.89 (11.85–19.43)	17.85 (13.06–21.56)	+3.82 (−0.37–7.08)	0.003	0.02
CD4+CCR4+CD45RO+	65.03 (59.52–72.07)	66.43 (61.31–74.79)	+0.50 (−5.79–6.98)	1	65.73 (56.81–72.67)	69.99 (65.23–77.56)	+5.86 (1.00–16.46)	0.003	0.011
CD4+CD25+CD45RA+	28.76 (20.95–39.98)	30.19 (23.73–39.65)	−0.80 (−8.39–3.15)	0.522	39.71 (29.77–44.41)	35.89 (19.59–41.02)	−3.76 (−13.51–2.30)	0.018	0.193
CD4+CD25+CD45RO+	49.80 (38.49–62.09)	49.82 (42.48–59.20)	+4.67 (−6.18–8.87)	0.416	44.76 (33.99–51.10)	48.32 (41.49–65.68)	+12.56 (4.70–18.53)	0.0006	0.003
CD4+CD25+CCR4+ CD45RO+	70.63 (64.65–77.49)	72.53 (65.35–81.45)	+2.40 (−3.74–11.31)	0.237	68.36 (60.54–75.96)	75.77 (70.7–81.39)	+6.65 (2.83–12.86)	0.0007	0.083
CD4+CD25^high^+CCR4+CD45RO+	75.68 (67.80–85.28)	78.81 (68.20–83.74)	+3.97 (−4.42–7.85)	0.326	73.76 (63.43–82.79)	82.87 (77.06–89.13)	+7.88 (0.19–15.87)	0.001	0.045
CD3+CD4+CCR9+β7+	1.34 (1.01–1.94)	1.46 (1.16–2.05)	+0.21 (−0.44–0.40)	0.623	1.65 (1.21–2.35)	1.42 (1.06–2.13)	−0.33 (−1.06–0.09)	0.024	0.038
CD4+CD25+	8.82 (7.23–12.44)	9.11 (7.58–11.18)	−0.10 (−3.31–2.51)	0.839	9.15 (7.14–12.51)	9.88 (7.63–12.37)	+1.37 (0.01–3.06)	0.012	0.1
CD4+CD25^high^	3.93 (3.10–5.34)	4.30 (3.12–4.96)	+0.08 (−1.41–1.55)	0.867	4.04 (2.99–5.21)	4.63 (3.43–5.54)	+1.06 (−0.27–1.7)	0.008	0.103
Total									
CD3+CD4+	14.95 (10.80–20.37)	11.75 (9.27–20.92)	−3.01 (−8.31–5.96)	0.229	14.04 (10.03–25.67)	13.35 (8.18–19.15)	−4.07 (−9.65–1.14)	0.039	0.668
CD3+CD4+CD62L^low^+	2.42 (1.47–3.53)	1.85 (1.46–3.03)	−0.67 (−1.47–0.56)	0.333	2.56 (1.78–4.19)	2.06 (1.28–3.06)	−0.83 (−1.91–0.49)	0.051	0.456
CD3+CD8+CD62L^low^+	2.53 (1.53–3.42)	2.43 (1.71–3.31)	−0.10 (−0.98–0.87)	0.792	2.70 (1.74–4.19)	2.02 (1.22–2.89)	−0.85 (−1.52–0.30)	0.014	0.088
CD3+CD8+CD45RA+ CD45RO-	5.23 (3.50–8.59)	5.79 (3.73–7.99)	−0.26 (−2.06–2.70)	0.402	5.57 (3.65–9.36)	4.70 (3.40–6.19)	−1.71 (−4.96–1.27)	0.031	0.195

## Supplementary Materials

The following are available online at https://www.mdpi.com/2072-6643/11/8/1925/s1, File S1: The study protocol, File S2: Consort 2010 checklist, File S3: Raw research data, File S4: Raw research data; Figure S1: Consort flow diagram.
