# Supplementary material for: Effects of Lactobacillus plantarum and Lactobacillus paracasei on the Peripheral Immune Response in Children with Celiac Disease Autoimmunity: A Randomized, Double-Blind, Placebo-Controlled Clinical Trial
Source: Nutrients. 2019 Aug 16;11(8):1925. doi: 10.3390/nu11081925 (PMC6723580; doi:10.3390/nu11081925)
Supplement: Supplementary file 1 [file nutrients-11-01925-s001.zip › Supplementary materials/Fig S1 Consort flow diagram.doc]

**CONSORT 2010 Flow Diagram**

**Allocation**

**Analysis**

**Follow-Up**

**Enrollment**

Assessed for eligibility (n=118 )

Excluded (n=29)

♦  Not meeting inclusion criteria (n=1)

♦  Declined to participate (n=28 )

♦  Other reasons (n=0 )

Analysed (n=40)
♦ Excluded from analysis (give reasons) (n=0)

Lost to follow-up (give reasons) (n=0)

Discontinued intervention (low compliance with study product, no reason given) (n=6)

Allocated to intervention **Probiotics** (n=46)

♦ Received allocated intervention (n=46)

♦ Did not receive allocated intervention (give reasons) (n=0)

Lost to follow-up (give reasons) (n=0)
Discontinued intervention (low compliance with study product, symptoms, refused blood draw) (n=5)

Allocated to intervention **Placebo** (n=43)

♦ Received allocated intervention (n=43)

♦ Did not receive allocated intervention (give reasons) (n=0)

Analysed (n=38)
♦ Excluded from analysis (give reasons) (n=0 )

Randomized (n=89)
