# Supplementary material for: Effects of Lactobacillus plantarum and Lactobacillus paracasei on the Peripheral Immune Response in Children with Celiac Disease Autoimmunity: A Randomized, Double-Blind, Placebo-Controlled Clinical Trial
Source: Nutrients. 2019 Aug 16;11(8):1925. doi: 10.3390/nu11081925 (PMC6723580; doi:10.3390/nu11081925)
Supplement: Supplementary file 1 [file nutrients-11-01925-s001.zip › Supplementary materials/File S1 Study protocol.doc]

**Detailed research Plan for CiPP	2012-01-16**

**Celiac disease Prevention with Probiotics – CIPP**

**Principal Investigator**
Daniel Agardh, MD, Associate professor
Mobile +46 768-870387
E-mail: [daniel.agardh@med.lu.se](mailto:daniel.agardh@med.lu.se)

**Co-workers**
Carin Andrén Aronsson, dietician
Sara Björck, MD
Charlotte Brundin, biomedical scientist

**Affiliations**
Unit for Diabetes and Celiac disease, Department of Clinical Sciences, Malmö
Lund University
Clinical Research Center
Malmö
205 02 Malmö

**Other collaborators**
Niklas Larsson, Head of Research
Gunilla Önning, Senior scientist
Probi AB, Ideon Gamma 1
Sölvegatan 41
223 70 Lund

Göran Molin, professor
Department of Food Technology - Engineering and Nutrition
Kemicentrum, Lund University
221 00 Lund

**Background**

Celiac disease is a chronic autoimmune disorder of the small intestine that affects up to 3% of children in Sweden. Most people diagnosed with the celiac disease have no or diffuse symptoms that make it difficult to detect, so called silent celiac disease, why study the disease often occurs through screening. Celiac disease is strongly associated with the presence of autoantibodies directed against tissue transglutaminase, also called tissue transglutaminase autoantibodies (TGA) that occurs in more than 90% of patients with celiac disease and lacking in healthy individuals (Dieterich et al, 1997). Celiac disease is also clearly linked to inherited HLA risk genes on chromosome 6, where 95% of all patients with celiac disease carry the haplotypes DQA1 * 0501-DQB1 * 0201 (DQ2) and 5% DQA1 * 0301 DQB1 * 0302-(DQ8) (Sollid et al, 1989).

The microbiota composition of the gut can affect both the intestinal barrier properties as well as the immune system's response to various dietary components. An unfavorable composition of the intestinal flora can turn parts of the immune system involved in chronic inflammation in celiac disease and inflammatory bowel disease. Probiotics are defined as live microorganisms that when ingested in sufficient quantities provides a health effect (FAO/WHO). Administration of probiotics in your diet have documented effect on the regulation of the immune system in chronic inflammation of the intestine and have therefore been considered as preventive treatment for inflammatory bowel disease (Llopis et al, 2009). Probiotics has also been shown to inhibit the pro-inflammatory immune response triggered by gluten in celiac disease (Laparra et al., 2010). A recently published study concerning the regulation of autoimmunity in experimental animal studies, showing that a mix of three Lactobacillus strains, l. paracasei 8700:2 and l. plantarum Heal 9 and 19, has a synergistic effect with respect to the reduction of the pro-inflammatory cytokine mediated immune response by stimulation of regulatory T-cells (Lavasani et al, 2009). Previous studies have also shown that Lactobacillus strains affect the permeability in the small intestine (Eun et al., 2010). However, there are no known prevention studies carried out on humans showing that administration of probiotics can influence the development of celiac disease. This requires prospective randomized studies that follows individuals with hereditary risk for celiac disease over time before they developed the disease (Nova et al., 2010).

Celiac disease Prediction in Skåne (CiPiS) study is a prospective study whose purpose is to investigate the prevalence of celiac disease in children with and without HLA risk (Björck et al., 2010). In the first part of the study 3.435 children (1.620 children with HLA-risk, 1.815 without HLA risk) was screened for TGA at 3 years of age and a second part was carried out and screened children at the age of 9. In the first study at 56/1.620 (3.5%) children was found to have biopsy confirmed celiac disease. Another 15/71 (21%) of the children were biopsied and was found to have normal small intestine mucosa in spite of increased levels of TGA. Despite the fact that these children are positive for TGA in repeated samples, they have not yet developed the disease. Children with TGA without biopsy confirmed celiac disease is defined as individuals with the potential risk of developing celiac disease later in life (so-called "latent celiac disease). These children are therefore followed by the Pediatrics clinic at the University hospital (Malmö) with regular checks about every 3 years.

The Environmental Determinants of Diabetes in the Young (TEDDY) study is an international study with the aim to investigate genetic, immunological and non-genetic environmental factors linked to development of type 1 diabetes (Rewers et al., 2010). Type 1 diabetes and celiac disease share several risk genes and therefor are the children regularly examined from the age of two with annual sampling for TGA to detect celiac disease. In this study, about 15% of children with TGA had a normal small intestine mucous membrane at the time of the survey, and these are followed by repeated blood samples every 3 to 6 months. The study is a multicentre study and includes research centers in Finland, Sweden, Germany and 3 states in the United States of America (Colorado, Washington and Georgia). In TEDDY, TGA was found in 11.2% of the children of which 4.7% was later diagnosed with celiac disease in Sweden compared with 7.5% and 2.1% in Finland. The reason for the difference between Sweden and Finland is unexpected because both countries are estimated to have equally high incidence of celiac disease in the general population. A difference has previously been demonstrated in TEDDY is that Finnish children to a greater extent than the corresponding Swedish children, are given supplemental probiotics.

**Scientific research question**

The study aim is to examine whether the oral ingestion of a mix of two different Lactobacillus strains of the species Lactobacillus paracasei and Lactobacillus plantarum can affect the autoimmune process of celiac disease reflected as a change of levels of TGA. Our hypothesis is Lactobacilli can directly regulate the autoimmune process in the lining of the small intestine by stimulation of regulatory T-cells, but also by influencing the permeability of thin intestinal mucous membrane of gluten peptides that stimulates specific T-cells.

The specific research questions are:

1. Will daily consumption of Lactobacilli in the dose 1010 cfu (colony forming units)/day for 6 months versus placebo result in that levels of TGA drops in subjects with latent celiac disease?
2. Will daily consumption of Lactobacilli in the dose 1010 cfu (colony forming units)/day for 6 months versus placebo result in that lowered levels of regulatory T-cells (CD4+CD25+) in subjects with latent celiac disease?
A. **Study plan**
   a. The start of the study is scheduled for **2012-02-01**. A total of 100 children with untreated latent celiac disease will be included to the study. The requirements for inclusion to the CiPP-study is a child with low positive values for tTG antibodies in two consecutive blood samples. Children who have undergone a biopsy which turned out normal are also eligible for the study. Cut off values for positive TGA samples are IgA tTG <30 U and >1.3 (in TEDDY) and IgA-tTG and IgG-tTG > 4 U/mL (in CiPiS). Children enrolled in TEDDY will be selected by Daniel Agardh DY and by Sara Björck for CiPiS who then notifies the study coordinator Carin Andrén Aronsson for TEDDY and Charlotte Brundin for CiPiS. The family will receive an information letter and a consent form, which should be completed before enrollment to the study. The forms are sent to the Unit of Diabetes and Celiac disease at Clinical Research Center (CRC) in Malmö and registered in the local database. Children who will participate in the study, will be contacted for scheduling a visit at the research clinic. Together with a written confirmation of the time for clinic visit, anesthetic cream (EMLA) and instruction before blood sampling will be sent home to the parents. When the written informed consent is obtained further planning for study participation in TEDDY to be coordinated between Carin Andrén Aronsson and the research nurses at the TEDDY clinics. Charlotte Brundin (biomedical scientist) and Marie Andersson-Turpeinen (research nurse) will co-ordinate children from the CiPiS study. All children enrolled to CiPP will be referred to one of the TEDDY clinics located in Malmö, Helsingborg or Kristianstad. Half of the study participants randomized into the group with daily intake of a preparation containing Lactobacilli (daily for 6 months) and the other half will be randomized into the placebo group.
   b. Randomization into each group takes place through a double blind procedure. Children enrolled in TEDDY and CiPiS are identified with a local-ID (serial number). Collected samples (blood, stool) and questionnaires are marked with the subjects TEDDY- or CiPiS id-number and date of sampling. Samples from subjects enrolled in CiPiS are also tagged with with Diabetes Prediktion i Skåne (DiPiS) study material in Region Skåne's quality controlled biobank, Biobank title 31 (H Elding L). The study number (i.e. DiPiS number) for CiPiS participants consists of 6 or 7 digits where the first digit corresponds to the maternity hospital baby is born and the last digit is number in sequence at multiple pregnancies, other figures is consecutively selected. The number in DiPiS is given to the child in connection with the child’s birth and enrolment into the screening study (DiPiS) for which CiPiS is based on. The data file with the mother's social security number, birth date and address, together with DiPiS-code exists in a separate file with limited availability. Responsible for this database is Peter Almgren (biostatistician). This data file will not be combined with other data files in DiPiS-database where signed informed consent, analysis results, and questionnaires from CiPiS are recorded.
   c. Probi AB is responsible for labeling the study material with a unique serial number on each packet (study ID for CiPP). When the informed consent was signed by the parents for participation in the study, the child's study ID will be added to the CiPP database by Carin Andrén Aronsson, who notifies the nurses at each clinic. The nurses will in turn notify Carin of what CiPP-id given to the child at the first visit.
   d. At the first visit, a total of 5 ml of venous blood and 5 ml serum samples will be collected. Sampling tubes for stool collection are handed out. The visit at the clinic will take approximately 30 minutes. Following visits will be completed at 3 and 6 months after the start in the study (see fig. 1).
   e. Stool samples collected at home should be kept chilled in a cooler (or frozen if stored for a longer time) until samples are delivered to the study nurse at the respective TEDDY clinic. For other sample handling for each clinic, please see fig. 2.
B. **Methods for analyzing TGA**
   a. Serum is prepared and analyzed by Charlotte Brundin (Biomedical scientist) at the CRC laboratory. The results are double-checked and signed by Daniel Agardh, which announces the results subsequently to Carin Andrén Aronsson (study coordinator) who will add them into the database. The method of measurement for TGA (RBA, radioligand binding assay) have been described in detail (Agardh et al, 2004). The IgA and IgG-TGA TGA are measured at separate times. In summary, synthesized human recombinant tissue transglutaminase (tTG) by in vitro transcription and translation together with radio-labelled methionine (Perkin Elmer LifeSciences, Inc., Boston, MA, United States). Patient serum is administered and tTG/antibody complex is separated with goat anti human IgA Agarose (Sigma, St. Louis, MO, United States). Radioactive marked tTG is then measured in a Wallace MicroBeta machine and is expressed in the unit U/ml. Limit value for positive test based on previous survey of 398 healthy blood donors.
C. **HLA-DQB1 genotyping**
   a. HLA-typing is already carried out in the two respective studies DiPiS and TEDDY and can be found in the respective database. HLA-typing has previously been performed using PCR and hybridization with allele specific probes for genotyping of HLA-DQA1 and DQB1 (Larsson et al, 2005)
D. **Flow cytometry**

### a. Charlotte Brundin receives the venous blood samples in the CRC laboratory and with reduced spin up produce peripheral blood mononuclear cells (PBMCS). PBMCS marks directly with a set of fluoro chrome conjugated antibodies against the following markers: CD3 (FITC-conjugated, clone SK7), CD4 (PerCP-conjugated, clone SK3), CD8 (PE or PerCP-conjugated, clone SK1), CD16 (PE-conjugated, clone SK7, 1), CD19 ( PerCP-conjugated, clone 4G7), CD25 (FITC-conjugated, clone the M-A251), CD62L (APC-conjugated, clone DREG-56), CD45RA (HI100, PE-conjugated clone), CD45RO (APC-conjugated, clone UCHL1), CD56 (PE-conjugated, clone MY31), CCR4 (PE-conjugated, clone 1GI), Integrin B7 (PE-conjugated, clone FIB504) and IgG2a (FITC-conjugated, clone X 39 series) and IgG1 (PE-conjugated, clone x 40) (Becton-Dickinson/PharMingen, CA, United States) and CCR9 (APC-conjugated, clone 248621, R&D Systems, Minneapolis, MN, United States).Proverna analyseras sedan med flödescytometri i en FACScalibur® (Becton Dickinson, CA, USA) och analyseras i CellQuest® software (Becton-Dickinson, CA, USA). Isotype matched antibodies are used as controls to set boundaries in a dot-plot quadrant and the lymphocyte populations are calculated as a percentage.

E. **Lactobacillus preparation**
   a. Probi AB is responsible for the Lactobacillus preparation. The active product included dried *Lactobacillus plantarum* Heal 9 and Lactobacillus *paracasei* 8700:2 (1x1010 cfu/day) and maltodextrin while the placebo product only contains maltodextrin. The products should be stored in a refrigerator and eaten in the morning with a meal. The study product is supplied by Probi AB. Each study product is labelled with the name of the study, the best before date, how they should be stored, subject number, manufacturer and the name of the principal investigator including his phone number.
F. **Analysis of the bacterial flora**
   a. Stool samples will be collected from the respective study clinic by Carin Andrén Aronsson one time per month. Stool samples are stored at -20°C in a freezer at Wallenberg laboratory, in before analysis. Probi AB retrieves all stool samples; store them in -80°C in time for analysis. To be able to follow that the given lactobacillus have established themselves in the intestine and as a measure of that study, the stool samples will be analyzed for the specific lactobacillus. DNA from stool samples are extracted with QIAamp DNA Stool Mini kit (Qiagen) according to the manufacturer's instructions, after previous double-rinse in PBS. The purity and quality of the extracted DNA is controlled with SmartSpec Plus spectrophotometer (Bio-Rad). To analyze the amount of L. plantarum and L. paracasei in stool samples the RT-Q-PCR analysis is used according to Haarman (2006) for optimization. Primers and probes are from Applied Biosystems. DNA extracted from pure cultures of L. plantarum Heal 9 and L. paracasei 8700:2 are used as reference checks.
   b. Analysis of bacteria’s in the stool samples are made with "Terminal Restriction Fragment Length Polymorphism (T-RFLP) of the 16S rRNA gene (16S rRNA gene is unique to bacteria and are available in different versions in all kinds of bacteria). Via biometric evaluation by means of multivariate data analysis, specific groups of bacteria’s (visualized as tops in a T-RFLP) are traced with specific physiological test results to examine if a correlation exists. This can then be verified by conventional univariate statistical methods. T-RFLP is also a respectable method for measuring bacterial diversity (diversity). T-RFLP is a PCR-based methodology as previously optimized, evaluated and used on stool samples from children (Wang et al., 2004; Wang et al., 2008). Depending on the results, we can also use cloning and sequencing of 16S rRNA genes for ensuring identification (Wang et al., 2004; Wang et al., 2005; Falk et al., 2007). Quantitative PCR for a number of selected bacterial groups of physiological or pathological interest will be made on all samples (Karlsson et al. 2011).
G. **Adverse events**
   a. Any deviation or suspected adverse event in the study must be registered by the responsible research nurse in children participating in the TEDDY study and by Sara Bjöck in children involved in the CiPiS study, using a specific form (see attachement 1). All registered incidents should be notified to principal investigator Daniel Agardh.
   b. In case of a suspected serious adverse event where ingestion of the product cannot be ruled out to be the cause, the same procedure shall be made as provided in paragraph (a). Daniel Agardh should then consider whether to open the code key. The code key is stored in a sealed envelope locked up in a safety box in the office of Daniel Agardh (Pediatric clinic, entrance 108, level 2, Skåne University Hospital) in Malmö.

**Significance of the study**
Celiac disease is one of the most common chronic diseases affecting children. Most children with the disease goes undetected if no screening is carried out. The only known treatment is a life-long gluten-free diet, but there is no cure for the disease. The downside of screening is the knowledge of being carriers of TGA without being affected by the disease. However, these children are at risk of developing the disease in the end, but there is currently no preventive method to stop the development process towards disease other than to start a life-long gluten-free diet. The hypothesis is that the intake of Lactobacilli can affect the autoimmune process in the gut by reducing the TGA levels and lower levels of regulatory T cells. The profit of the study is both a better understanding of certain lactobacillus influence of autoimmunity in latent celiac disease and, on the other hand, a hope to reduce the risk of developing celiac disease manifests.

**References**

Agardh D, Carlsson A, Lynch K, Axelsson I, Lemmark A, Ivarsson SA (2004). [Using radioligand-binding assays to measure tissue transglutaminase autoantibodies in young children.](http://www.ncbi.nlm.nih.gov/pubmed/15456194) *Acta Paediatr*. Aug;93(8):1046-51.

Björck S, Brundin C, Lörinc E, Lynch KF, Agardh D (2010).[Screening detects a high proportion of celiac disease in young HLA-genotyped children.](http://www.ncbi.nlm.nih.gov/pubmed/19915493) *J Pediatr Gastroenterol Nutr*. Jan;50(1):49-53.

Dieterich W, Ehnis T, Bauer M, Donner P, Volta U, Riecken EO, Schuppan D (1997. [Identification of tissue transglutaminase as the autoantigen of celiac disease.](http://www.ncbi.nlm.nih.gov/pubmed/9212111) Nat Med. Jul;3(7):797-801.

Eun CS, Kim YS, Han DS, Choi JH, Lee AR, Park YK. (2010) [Lactobacillus casei prevents impaired barrier function in intestinal epithelial cells.](http://www.ncbi.nlm.nih.gov/pubmed/21143526) APMIS. 2011 Jan;119(1):49-56.

Falk, A., Olsson, C., Ahrné, S., Molin, G., Adawi, D. and Jeppsson, B. (2007). Ileal pelvic pouch microbiota from two former ulcerative colitis patients, analysed by DNA-based methods, were unstable over time and showed the presence of *Clostridium perfringens* group. *Scandinavian Journal of Gastroenterology* 42: 973-985.

Karlsson, C.L.J, Molin, G., Cilio, C.M. & Ahrné, S. (2011). The pioneer gut microbiota in human neonates vaginally born at term - a pilot study. *Pediatric Research* (*accepted for publication*).

Laparra JM, Sanz Y (2010). [Bifidobacteria inhibit the inflammatory response induced by gliadins in intestinal epithelial cells via modifications of toxic peptide generation during digestion.](http://www.ncbi.nlm.nih.gov/pubmed/20052669) *J Cell Biochem*. Mar 1;109(4):801-7.

Larsson HE, Lynch K, Lernmark B, Nilsson A, Hansson G, Almgren P, Lernmark A, Ivarsson SA; DiPiS Study Group (2005) Diabetes-associated HLA genotypes affect birthweight in the general population. *Diabetologia* 48(8):1484-91.

Lavasani S, Dzhambazov B, Nouri M, Fåk F, Buske S, Molin G, Thorlacius H, Alenfall J, Jeppsson B, Weström B (2010). [A novel probiotic mixture exerts a therapeutic effect on experimental autoimmune encephalomyelitis mediated by IL-10 producing regulatory T cells.](http://www.ncbi.nlm.nih.gov/pubmed/20126401) *PLoS One*. Feb 2;5(2):e9009.

Llopis M, Antolin M, Carol M, Borruel N, Casellas F, Martinez C, Espín-Basany E, Guarner F, Malagelada JR (2009).[Lactobacillus casei downregulates commensals' inflammatory signals in Crohn's disease mucosa.](http://www.ncbi.nlm.nih.gov/pubmed/18839424) *Inflamm Bowel Dis*. Feb;15(2):275-83.

Nova E, Pozo T, Sanz Y, Marcos A (2010). [Dietary strategies of immunomodulation in infants at risk for celiac disease.](http://www.ncbi.nlm.nih.gov/pubmed/20579407) *Proc Nutr Soc*. Aug;69(3):347-53. Epub 2010 Jun 28.

Sollid LM, Markussen G, Ek J, Gjerde H, Vartdal F, Thorsby E (1989). [Evidence for a primary association of celiac disease to a particular HLA-DQ alpha/beta heterodimer.](http://www.ncbi.nlm.nih.gov/pubmed/2909659) *J Exp Med.* Jan 1;169(1):345-50.

Rewers M, TEDDY Study Group (2008). [The Environmental Determinants of Diabetes in the Young (TEDDY) Study.](http://www.ncbi.nlm.nih.gov/pubmed/19120261) *Ann N Y Acad Sci*. Dec;1150:1-13.

Wang, M., Ahrné, S., Antonsson, M. and Molin, G. (2004). T-RFLP combined with principal component analysis and 16S rRNA gene sequencing: an effective strategy for comparison of faecal microbiota in infants of different ages. *Journal of Microbial Methods* 59: 53-69.

Wang, M., Ahrné, S., Jeppsson, B. and Molin, G. (2005). Comparison of bacterial diversity along the human intestinal tract by direct cloning and sequencing of 16S rRNA genes. *FEMS Microbial Ecology* 54: 219-231.

Wang, M., Karlsson, C., Olsson, C., Adlerberth, I., Wold, A., Strachan, D.P., Martricardi, P.M., Åberg, N., Perkin, M.R., Tripodi, S., Coates, A.R., Hesselmar, B., Saalman, R., Molin, G. and Ahrné, S. (2008). Reduced diversity in the early fecal microbiota of infants developing atopic eczema: Low diversity in early microbiota of infants developing atopy. *Journal of Allergy and Clinical Immunology* 121: 129-134.

Fig. 1 Schematic overview of the study participants visiting schedule in the CiPP-study.

Fig. 2. Handling of samples received in the CiPP study, at respective research clinics

| **Clinic** | **Blood samples** | **Stool samples** | **Study material** |
| --- | --- | --- | --- |
| Malmö | Blood draw by RN. RN notifies Charlotte (CB) that a blood sample is collected. CB collects, analyses and store samples in freezer (CRC, Building 91:1).  Test results are registered in database by CAA | RN receives samples.  Stool samples are stored in a freezer at WB. Probi AB is responsible for collecting,  analysis and storage | RN distributes study material and collects leftover material  Randomization ID is notified to CAA |
| Helsingborg | Blood draw by RN.   Lab personal are responsible for sample transfer to CB. | RN receives samples, stored in a local freezer at the clinic.  CAA warrants that samples are transported to the central freezer on WB Malmö | RN distributes study material and collects leftover material  Randomization ID is notified to CAA |
| Kristianstad | Blood draw by RN.   Lab personal are responsible for sample transfer to CB. | RN receives samples, stored in a local freezer at the clinic.  CAA warrants that samples are transported to the central freezer on WB Malmö | RN distributes study material and collects leftover material  Randomization ID is notified to CAA |

CAA=Carin Andrén Aronsson; CB=Charlotte Brundin; WB=Wallenberg laboratory in Malmö; RN=research nurse.
